# The Skin, Its Correlations and Functions

**Published:** 1887-03

**Authors:** F. G. Welsh

**Affiliations:** New York City


### THE SKIN—ITS CORRELATIONS AND FUNCTIONS.

It is a low, incorrect and unworthy view of this grand organ to regard it only and simply as a protective covering to the body. It is in truth much more—A LIVING, SENSITIVE, BREATHING, ABSORBING, EXCRETING MEMBRANE OF EXQUISITE STRUCTURE AND ENDOWMENTS. Herein many of the prime operations of life take place. The skin may truly be called a great appendage to the heart and lungs—being an equal co-worker with them in the circulation of the blood. It not only rids the blood of its carbon and supplies it with oxygen, but regulates its density, evaporating its watery constituents. The skin is at once the grand drying, draining and ventilating apparatus of the body. It is in itself an universally expanded lung, kidney, liver, heart and bowels! It is the greatest medium of nervous and vascular expansion, and therefore possesses sensibilities of exquisite and tactile endowments. Altogether the skin is an admirable piece of design, illustrating alike the wisdom and the goodness of the Supreme Architect.

On the sound condition of this organ as much as, if not more, than that of any other, depends the comfortable working of the living machinery. On it, are reflected *their* ailments, and its derangements, in turn, are sure materially to modify for the worse the play of the interior apparatus. Herein is apparent how potent, not to say how safe a battery the skin presents for the reduction of disease. In fact, many acute and chronic maladies select the skin, as it were, as the common sewer for the running off of morbid elements which have accumulated in the system, and which no over-action of the bowels or kidneys has been of avail to eliminate.

Everybody knows that, in many diseases, THE BATTLE IS LOST OR WON ON THE FIELD OF THE SKIN; according as its safety-valve functions rise or fall.

It is the principle outlet of the body. Four times more matter is carried out of the body by the cutaneous surface every day than by the alimentary canal.

It is a complete web of nerves and blood-vessels; its thickly studded pores constitute the vastest system of corporeal drainage. Twenty-eight miles of tubing, seven million pores. If these become partially closed, disease is certain. It is pre-eminently fitted and intended to be the battle-ground of the physician in his conflict with disease.—Extract from *Treatise* by F. G. WELSH, M. D., of New York City.

